# Physical activity as an aid to smoking cessation during pregnancy: Two feasibility studies

**DOI:** 10.1186/1471-2458-8-328

**Published:** 2008-09-23

**Authors:** Michael Ussher, Paul Aveyard, Tim Coleman, Lianne Straus, Robert West, Bess Marcus, Beth Lewis, Isaac Manyonda

**Affiliations:** 1 Division of Community Health Sciences, St. George's University of London, London, SW17 0RE, UK; 2 Department of Primary Care and General Practice, University of Birmingham, Edgbaston, B15 2TT, UK; 3 School of Community Health Sciences, Division of Primary Care, Queen's University Medical Centre, Nottingham, NG7 2UH, UK; 4 Barcelona Centre for International Health Research (CRESIB), Hospital Clínic de Barcelona, Universitat de Barcelona, Rosselló 132, 4a planta, 08036 Barcelona, Spain; 5 Cancer Research UK Health Behaviour Research Centre, Department of Epidemiology and Public Health, University College London, 2-16 Torrington Place, London, WC1E 6BT, UK; 6 Department of Community Health and Department of Psychiatry and Human Behavior, Brown University, 121 South Main Street, Providence, Rhode Island, 02903, USA; 7 School of Kinesiology, University of Minnesota, 209 Cooke Hall, 1900 University Ave, Minneapolis, MN 55455, USA; 8 Department of Obstetrics and Gynecology, St George's Hospital, Blackshaw Road, London, SW17 0QT, UK

## Abstract

**Background:**

Pharmacotherapies for smoking cessation have not been adequately tested in pregnancy and women are reluctant to use them. Behavioural support alone has a modest effect on cessation rates; therefore, more effective interventions are needed. Even moderate intensity physical activity (e.g. brisk walk) reduces urges to smoke and there is some evidence it increases cessation rates in non-pregnant smokers. Two pilot studies assessed i) the feasibility of recruiting pregnant women to a trial of physical activity for smoking cessation, ii) adherence to physical activity and iii) womens' perceptions of the intervention.

**Methods:**

Pregnant smokers volunteered for an intervention combining smoking cessation support, physical activity counselling and supervised exercise (e.g. treadmill walking). The first study provided six weekly treatment sessions. The second study provided 15 sessions over eight weeks. Physical activity levels and continuous smoking abstinence (verified by expired carbon monoxide) were monitored up to eight months gestation.

**Results:**

Overall, 11.6% (32/277) of women recorded as smokers at their first antenatal booking visit were recruited. At eight months gestation 25% (8/32) of the women achieved continuous smoking abstinence. Abstinent women attended at least 85% of treatment sessions and 75% (6/8) achieved the target level of 110 minutes/week of physical activity at end-of-treatment. Increased physical activity was maintained at eight months gestation only in the second study. Women reported that the intervention helped weight management, reduced cigarette cravings and increased confidence for quitting.

**Conclusion:**

It is feasible to recruit pregnant smokers to a trial of physical activity for smoking cessation and this is likely to be popular. A large randomised controlled trial is needed to examine the efficacy of this intervention.

## Background

In the UK, 17% of women admit to smoking throughout pregnancy [1], but this is likely to be an underestimate since these data were collected retrospectively and many pregnant women that smoke deny it [2]. Smoking during pregnancy is harmful to the fetus, mother, and the child after birth. It is a major preventable contributor to miscarriage and stillbirth, being associated with 4000 deaths annually, as well as causing low birth weight with its attended risks [3,4]. The risks to the mother, in addition to the long term risks reported for smokers in general, include placental abruption [5,6]. The newborns of smokers have a higher perinatal mortality and are at an increased risk of neo-natal mortality, sudden infant death syndrome, learning difficulties in childhood, problem behaviour, asthma and childhood obesity [7-11].

In the UK approximately half of pregnant smokers stop for at least part of their pregnancy [1], but, of these, two thirds are likely re-start post-natally [12]. Women who smoke throughout pregnancy are more likely to come from economically disadvantaged backgrounds [13]. The most effective smoking cessation therapy in non-pregnant smokers combines behavioural support with nicotine replacement therapy (NRT), bupropion, nortriptyline, or varenicline [14-16]. Of these medications, only NRT has been licensed for use during pregnancy. A recent trial provides evidence that the addition of NRT to cognitive-behavioural therapy for pregnant smokers increases rates of smoking cessation [17]. However, there are concerns about NRT harming the foetus [18] and many women are reluctant to use NRT at all during pregnancy [19]. Behavioural support increases spontaneous smoking cessation rates in pregnancy by up to 7%, such that up to 15% of women who receive this abstain from smoking until the end of their pregnancy [20]. It would therefore seem that treatments that increase the effectiveness of behavioural support deserve concerted research efforts.

Most attempts to stop smoking, if unaided, end within a week [21] at least in part because of the strong urges to smoke experienced by those attempting to stop. Medications that are effective at smoking cessation reduce urges to smoke. A systematic review of short-term studies concluded that moderate intensity physical activity (equivalent to a brisk walk) reduced cravings in abstinent smokers [22]. When undertaken for at least 110 minutes per week during an eight week intervention, this activity has been associated with smoking cessation in non-pregnant women [23]. While there is no definitive evidence that physical activity improves abstinence rates, moderate physical activity is an attractive potential smoking cessation intervention in pregnancy because it is recommended [24] and might prove popular as it could prevent excessive weight gain [25]. There are no reported studies that have investigated physical activity for smoking cessation in pregnancy. Following recommendations for developing complex interventions [26], we conducted two uncontrolled, exploratory phase II studies investigating this topic. Our aim was to assess the feasibility of implementing a physical activity intervention for smoking cessation during pregnancy in terms of (i) recruitment, (ii) attendance, (iii) physical activity adherence and (iv) acceptability.

## Methods

This paper reports the results of two feasibility studies, referred to as Study 1 and Study 2. The studies were conducted sequentially, and Study 2 was a modified version of Study 1, based on the experience gained from Study 1.

### Participants

Pregnant women booking for antenatal care at one London hospital were eligible if they were aged 18 years and above, smoked at least one cigarette a day now and during the previous year, wanted to quit and had no contraindications to moderate intensity exercise [24,27]. We included women reporting such a low consumption of cigarettes because many pregnant smokers under-report the number of cigarettes they smoke [28]. Women were recruited between 12 and 20 weeks gestation and were not permitted to use NRT. Women were eligible irrespective of their current levels of physical activity.

### Recruitment

Women's antenatal smoking status is recorded on the hospital computerised patient administration system (PAS). For Study 1 all the women were recruited by telephoning smokers identified via the PAS, a recruitment strategy previously shown to be effective [29]. However, several women were surprised and unhappy about being 'cold called'. Therefore in Study 2, accompanying the standard letter inviting women for an antenatal appointment we included a letter of invitation to smokers to join the study, warning women about the telephone call. The study was also advertised on posters in the antenatal clinics and via talks given to women in the nuchal scan clinics. Recruitment was supplemented by referral from hospital and community midwives trained to discuss smoking during pregnancy. Both studies were approved by the Wandsworth Local Research Ethics Committee. All participants provided written informed consent.

### Smoking cessation and physical activity interventions

Participants received pregnancy-specific behavioural support and self-help guides for smoking cessation [30,31]. Behavioural support covered the importance of avoiding lapses, managing withdrawal symptoms and urges to smoke, enhancing self-confidence and relapse prevention [32,33]. A therapist trained to NHS standards [34] prepared women for cessation and all women stopped abruptly on their quit day. The same member of staff delivered the intervention and made the research assessments.

In Study 1 there were six weekly sessions of supervised exercise and in Study 2 there were two supervised sessions for six weeks and one supervised session for three weeks. The supervised exercise involved women exercising at moderate intensity, measured by the Rating of Perceived Exertion ('fairly light' to 'somewhat hard') [35], progressing across the sessions to around 30 minutes of exercise. For Study 1, women walked in the local area accompanied by the researcher, or followed an antenatal exercise DVD involving light callisthenic and cardiovascular-type exercises. Poor weather was sometimes a deterrent to walking and some women said that the DVD exercises were not sufficiently taxing. Therefore, for Study 2 for one of the two weekly sessions (conducted at the hospital) women used a stationary cycle (Monark 824E) or a treadmill (Lifefitness TR500HR) during which they could watch television while exercising. The second session each week took place at home or at the hospital, where women followed an exercise DVD, went for a walk in the local area or used the previously stated exercise equipment.

Physical activity counselling was employed to increase the physical activity women took outside of the supervised exercise. Standard cognitive-behavioural techniques were employed, including goal setting, self-monitoring, decision-balance sheets and relapse prevention plans [36]. The counselling protocol had been used successfully to increase activity levels in a previous trial [37]. The women were encouraged to use physical activity as a strategy to reduce cigarette cravings and withdrawal [22]. Women were advised to be active for continuous periods of at least 10 minutes for at least 110 minutes each week [23] progressing towards accumulating 30 minutes of moderate intensity activity on five or more days a week [24]. The emphasis was on walking, which is popular among pregnant smokers [38,39]. A home-based antenatal exercise DVD (YMCA) and booklet were also provided [40]. In order to promote physical activity adherence the women in Study 2 were given a Digi-Walker SW-200 pedometer [41] and were advised to progress towards 10,000 steps each day [42].

### Research design and procedure

Both Study 1 and Study 2 were uncontrolled and in both cases participants attended individual treatment sessions. For Study 1 treatment sessions began one week prior to participants' attempts at smoking cessation and continued to four weeks after their quit day. Participants attended six weekly sessions each with 15 minutes of smoking cessation support, 15 minutes of physical activity counselling, and 20–30 minutes of supervised exercise.

For Study 2 the treatment sessions commenced two weeks before women's quit dates and ended six weeks afterwards. Participants in Study 1 requested more supervised physical activity. Therefore, for Study 2, following the initial visit, women attended twice weekly for six weeks, then weekly for three weeks, totalling 15 appointments. During the first six weeks of treatment, one session each week involved 15 minutes of smoking cessation support and 20–30 minutes of supervised exercise. The other session involved 15 minutes of physical activity counselling and 20–30 minutes of supervised exercise. During the final three weeks, sessions consisted of physical activity counselling with supervised exercise only.

### Measures

In most cases, identical measures were used in Studies 1 and 2. At the first visit, demographics, smoking characteristics, nicotine dependence [43], confidence, importance, and determination to stop smoking were recorded [44]. Following quit day, at every visit, cigarettes smoked during the week were recorded and abstinence biochemically confirmed by expired air CO concentration (Bedfont Smokerlyzer) of less than <8 ppm [45]. If a participant did not attend we re-scheduled an appointment within 48 hours and women withdrawing from the programme were counted as having relapsed to smoking. In Study 2 only, for the two weeks before quitting and on the quit day, women rated their desire to smoke and well-being before and after supervised exercise [46]. This was used to reinforce the acute benefits of exercise.

During treatment and at the follow-up, physical activity was assessed by structured interview of seven-day recall [47] and bouts of moderate intensity activity lasting 10 minutes or more were recorded. This measure has adequate validity for pregnancy [48]. Attitudes to physical activity were assessed by the level of agreement with three items: 'Do you see yourself as the kind of person who exercises regularly?', 'Is the thought of exercising regularly something that you find appealing?' and 'Do you think exercising is something that you should do?'.

In both studies, we assessed the acceptability of the intervention by a 30 minute semi-structured interview with five abstinent women and five women who had relapsed. The women were interviewed at end-of-treatment and at eight months gestation. In addition, at the first visit all the women were asked 'Would you be willing to attend a trial of physical activity as an aid to smoking cessation if you had an equal chance of being assigned to a control group providing help with stopping smoking, but not promoting physical activity' (yes/no).

### Data analysis

All analyses were conducted using SPSS version 14. Changes in the desire to smoke and happiness scores between pre and post-exercise were assessed using analysis of variance (ANOVA). For the qualitative analysis, the interviews were recorded, transcribed and then analysed for emerging themes [49].

## Results

Overall, the number of smokers recorded in the Patient Administration System (PAS) in 2000 was 12.6% (542/4290) and in 2006 it was 7.8% (436/5673). During the two periods of recruitment a total of 3164 pregnancies were recorded and 8.8% (277) of these women were recorded as smoking. Combining both studies, 11.6% (32/277) of pregnant smokers recorded on the PAS were recruited. Of the women smoking who we were able to contact and invite to join the study, overall, 23% (32/140) were recruited. The participants claimed to smoke about 9 cigarettes per day prior to the intervention, which they said was about half their pre-pregnancy level of smoking. However, their exhaled carbon monoxide was compatible with a higher smoke intake than is typical from 9 cigarettes per day (Table 1). At baseline, less than a quarter of the women did 30 minutes of at least moderate intensity physical activity on five or more days each week, and walking was the main activity. While most women expressed a strong determination to stop smoking, only a few were confident that they could do so. Most recognised that physical activity was something they ought to be doing, but while the thought of doing so was appealing many did not identify themselves as the kind of person that exercised regularly.

**Table 1:** Baseline characteristics of the women in study 1 and study 2

**Variable**	**Study 1**	**Study 2**	**Overall**
	No. (%)	No. (%)	No. (%)
	N = 10	N = 22	N = 32
Married or living with partner	7 (70)	16 (72.7)	23 (71.8)
White European	9 (90)	14 (63.6)	23 (71.8)
Professional/managerial occupation	7 (70)	8 (36.4)	15 (46.9)
Employed	5 (50)	16 (72.4)	21 (65.6)
Desperately/very important to quit smoking	9 (90)	22 (100)	21 (96.9)
Extremely/very determined to quit smoking	9 (90)	19 (86.4)	28 (87.5)
'Extremely' or 'very' high rating of chances of giving up smoking	0	4 (18.1)	4 (12.5)
Walking as main exercise mode	20 (90.9)	9 (90.0)	29 (90.6)
Achieving 30 minutes of physical activity at least 5 days a week	3 (30.0)	4 (18.2)	7 (21.9)
Responded 'to some degree or very much so':			
See yourself as the kind of person who exercises regularly	No record	9 (40.9)	
Thought of exercising is appealing	No record	13 (59.1)	
Think exercise is something you should do	No record	21 (95.5)	
	**Mean (SD)**	**Mean (SD)**	**Mean (SD)**
Age	32.7 (5.3)	29.0 (5.6)	30.2 (5.7)
Full-time education (years)	14.4 (3.1)	14.0 (3.3)	14.2 (3.2)
Gestation (weeks)	16.5 (6.3)	16.2 (3.2)	16.3 (4.3)
Cigarettes per day now	10.9 (6.3)	8.2 (5.1)	9.03 (5.5)
Cigarettes per day before pregnancy	No record	18.4 (7.9)	
Fagerström Test of Nicotine Dependence score	3.6 (1.7)	3.2 (1.7)	3.3 (1.7)
Expired carbon monoxide level (ppm)	13.2 (5.6)	20.8 (13.0)	18.3 (11.6)
Reports of hours of physical activity per week	2.4 (1.2)	3.6 (2.8)	3.1 (2.5)
Days per week achieving 30 minutes of physical activity	2.7 (2.2)	2.9 (2.1)	2.8 (2.1)

Details of recruitment for the separate studies are given below:

### Study 1

Across a two month period in 2000, 42 women recorded as 'current smokers' on the PAS were telephoned and invited to join the study (Table 2). Six of these women (14%) reported that they were no longer smoking and were therefore ineligible. Of the 36 women still smoking, 19 women (53%) declined to take part; another seven women (7/36, 19%) passively refused by saying they would participate but did not appear for their first appointment. Ten of the women who were still smoking (10/36, 28%) took part in the study. Of the 10 recruits, two women did not attend the treatment session on their quit day and by four weeks after the quit day three women had relapsed; all of these women withdrew from the programme. The remaining five women were continuously abstinent from quit day to eight months gestation. For the five women who remained abstinent the mean (SD) expired CO value for their five assessments following quit day was 3.9 (1.1) ppm (range 2–6 ppm). For both studies, in all instances where the women reported smoking abstinence this was confirmed by an expired CO reading of <8 ppm. The five abstinent women in study 1 attended all the treatment sessions and the follow-up. Relative to baseline, these abstinent women increased moderate intensity physical activity by 0.7 hours one week after quit day, but the increase fell to 0.3 hours four weeks after quit day, and the women had a mean decline in physical activity of 1.4 hours at eight months gestation. Three of the five women achieved the minimum target of 110 minutes of activity at both one and four weeks. The time spent in supervised exercise was not recorded in Study 1.

**Table 2:** Smoking status recorded in patient administration system (PAS) at first antenatal booking visit and recruitment rates

	**Study 1**	**Study 2**	**Overall**
Total pregnancies recorded on PAS	734	2430	3164
	% (no.) out of total pregnancies	% (no.) out of total pregnancies	% (no.) out of total pregnancies
Quit smoking since pregnancy	18.9 (139/734)	25.2 (613/2430)	23.8 (752/3164)
Currently smokes ≥ one cigarette a day	11.0 (81/734)	6.6 (160/2430)	7.3 (232/3164)
Smokes occasionally	1.0 (8/734)	1.2 (28/2430)	1.1 (36/3164)
**Recruitment method**	% (no.) out of total smokers recorded	% (no.) out of total smokers recorded	% (no.) out of total smokers recorded
*(a) Direct telephoning*			
Invited to join study	47.2 (42/89)	66.0 (124/188)	61.9 (166/277)
Recruited	11.2 (10/89)	6.9 (13/188)	8.3 (23/277)
(b) Recruited via *flyer*	NA	1.6 (3/188)	
(c) Recruited via *presentation at nuchal scan clinic*	NA	0.6 (1/188)	
(d) Recruited via *posters*	NA	0	
*(e) Recruited via midwife referral*	NA	2.7 (5/188)	
**Total recruited**	11.2 (10/89)	11.7 (22/188)	11.6 (32/277)

### Study 2

Across a five month period in 2006 nine women recorded as 'current smokers' on the PAS were recruited through flyers, presentations or midwife referral (see Table 2). Across the same period, a further 124 women recorded as 'current smokers' on the PAS were telephoned and invited to join the study (see Table 2). Twenty (16%) of these women reported no longer smoking and were therefore ineligible. Of the 104 smokers, 66 (63%) declined to take part; another seven women (25/104, 24%) passively refused by not attending their first appointment. Thirteen of the women smokers who were telephoned (13/104, 13%) took part in the study. In total 24% (32/133) of those currently smoking joined the study.

Six women withdrew before quit day. After one week of smoking abstinence four of the original 22 women (18%) reported maintaining lapse-free abstinence, six women attended despite having smoked, and the remaining six women who did not attend were counted as having relapsed. At six weeks four women continued to maintain abstinence and four women attended who were smoking. By eight months of gestation 14% (3/22) of the women reported continuous abstinence from smoking and one woman who was smoking continued to attend. For the three women who reported abstinence the mean (SD) expired CO value for their seven assessments following quit day was 3.2 (1.2) ppm (range 1–7 ppm). All three women who maintained abstinence attended at least 13 (86.6%) of the 15 treatment sessions. Of the nineteen women recorded as having relapsed, eight confirmed that they had smoked (42%), five said that they were abstinent but had withdrawn because they could not attend due to other commitments, one woman became immobile with back problems, two women withdrew as they did not feel the intervention was helping, two women gave no reason for non-attendance and one woman could not be contacted.

Among the four abstinent women, mean physical activity rose by 42 minutes/week at one week after their quit day. Among the three women remaining abstinent through to eight months gestation, physical activity increased by a mean of 48 minutes/week at one week of abstinence, a mean of 3 hours 54 minutes/week at six weeks, and a mean of 3 hours at eight months gestation. All three achieved the minimum target of 110 minutes of activity after both one and six weeks. The mean (SD) minutes of supervised exercise recorded at the first visit (N = 22) was 8.4 (3.5), on the quit day (N = 16) it was 19.9 (8.6) minutes and five and six weeks after quitting (N = 6) it was 23.3 (6.1) and 18.5 (14.5) minutes, respectively.

Using analysis of variance, ratings of desire to smoke were significantly lower after a bout of supervised exercise, relative to before exercise, at one and two weeks before the quit day, and on the quit day following overnight abstinence (see Table 3). Additionally, compared with before exercise, ratings of happiness were significantly higher after a bout of supervised exercise at two weeks before the quit day, and on the quit day following overnight abstinence (see Table 3). 94% (30/32) of the women recruited said that they would like to join a study involving randomisation to a physical activity or control condition.

**Table 3:** Mean (SD) ratings of desire to smoke and happiness immediately before and after a bout of exercise

	Two weeks		One week			
	Before quit		Before quit		Quit day	
	(N = 21)	^a^P	(N = 15)	^a^P	(N = 14)	^a^P
Desire to smoke before exercise	2.5 (1.7)		1.8 (1.4)		3.6 (2.4)	
Desire to smoke after exercise	*1.6 (0.8)	0.001	*1.3 (0.8)	0.041	*2.2 (1.4)	0.021
Happiness before exercise	5.1 (0.8)		4.5 (1.4)		4.1 (1.5)	
Happiness after exercise	*5.5 (1.1)	0.031	5.0 (1.5)	0.056	*4.7 (1.5)	0.025
Mean (SD) minutes of supervised exercise	8.8 (3.1)		15.1 (5.8)		18.8 (8.6)	

^a^=P value for comparison between ratings before and after exercise, using analysis of variance.

*=Significant difference relative to before exercise at P < 0.05.

### Interview findings

Four main themes emerged from the interviews:

1) *The benefits of exercise included*:

- distraction and substitution (e.g. "I'm replacing smoking with exercise"),

- management of cravings/withdrawal symptoms ("I haven't being doing as much exercise recently and it's during that period that I'm feeling like those odd cigarettes.")

- weight management ("Now that I am doing more exercise I feel less worried about putting on weight.")

- being in control ("Exercise made me feel more capable and in control, which you could lose quite easily through pregnancy.")

- confidence for stopping smoking, increased energy, mood and self-esteem. The women said that physical activity helped them feel more like a non-smoker.

2) *Self-monitoring of physical activity levels*:

- Use of the pedometer and diary cards was popular and it was motivating ("You felt a real sense of achievement at the end of the day.")

- The pedometer caused some difficulty ("Once you start to get a bump the pedometer gets really uncomfortable and it digs in")

3) *Barriers to physical activity:*

- Lack of time due to home and work commitments was the most common barrier mentioned.

- Pregnancy related barriers, such as lack of energy, nausea, being less mobile, pregnancy complications, feeling self-conscious and vulnerable were also frequently reported.

4) *The intervention:*

- The demands of the intervention were generally acceptable ("The discipline of having to come twice a week kept it in your head all the time.")

- Some women in Study 2 found twice weekly appointments demanding, but they preferred attending hospital to having a home visit.

## Discussion

These two studies demonstrate, for the first time, that it is feasible to recruit pregnant smokers to exercise-based smoking cessation programmes. Our recruits were generally positive about both programmes, although the hospital-based scheme was viewed more positively than the programme delivered in women's homes. One quarter of the women recruited to either of the programmes quit smoking to the end of pregnancy, a rate which is similar to that shown for NRT in non-pregnant smokers [16]. However, this may be an optimistic estimate of the expected smoking cessation rates because we verified abstinence by expired CO rather than by a more reliable measure such as cotinine [50]. Moreover, the study did not include a control condition and quit rates might have been higher than usual had a control condition been used because these women were interested in increasing physical activity and therefore may have been more health conscious and more motivated to stop smoking than usual.

The proportion of women in the present study meeting government recommendations for physical activity (22%) was lower than the 36% reported in a recent survey of pregnant smokers [51]. This suggests that the current findings apply to reasonably sedentary pregnant smokers. Consistent with previous surveys of pregnant smokers, walking was the most popular form of activity [38,39]. The overall percentage of women recorded as smokers at their first antenatal booking visit in this study (9%) is markedly lower than national data which shows that 17% of women continue to smoke throughout pregnancy [1]. This may be partly due to the large proportion of South Asian women in the hospital's antenatal population (national smoking rates have been reported to be as low as 4% among Asian women) [52]. There was a reduction of around 5% in the number of smokers recorded in the PAS in 2006 compared with 2000. This is consistent with national surveys in the UK showing that the proportion of women smoking throughout pregnancy fell from 19% in 2000 to 17% in 2005 [1]. However, these reductions may partly reflect poor rates of enquiry or reduced disclosure as a result of the increased stigma associated with smoking [2,53]. Rates of disclosure might have been increased by presenting a multiple-choice question about smoking status [54]. The average level of cigarette consumption is consistent with national data [12].

Of those recorded as smokers at their first antenatal booking visit only 12% of the women were recruited to the two studies. In absolute terms this recruitment rate is low and it suggests that the intervention is unlikely to have a major impact on public health. However, the proposed intervention is still important since it is necessary to assess whether interventions for pregnant smokers are effective among those who accept them. We had not anticipated any higher recruitment rates, as according to UK statistics [1] half of all women stop smoking in pregnancy and nearly all of these do so prior to or early in pregnancy without any contact with antenatal care services and without formal support. We were therefore recruiting from the 'recalcitrant' half of women who are left, probably many of whom are reluctant to stop smoking and to receive help with stop smoking by any means. Thus, we are encouraged that our recruitment rate compares favourably with rates for dedicated stop smoking in pregnancy services of 5% for pregnant smokers in a national report [55], and 10% reported by the smoking cessation service in the same hospital from which we recruited. Consistent with a previous survey [51], our rate of recruitment suggests that the vast majority of pregnant women, including those from low-income groups, who are interested in receiving help with stopping smoking would be willing to join a study offering a physical activity intervention. Our findings also suggest that women would volunteer irrespective of whether they were allocated to the control group or the physical activity group. It noteworthy, that of the women recorded as smokers only around 60% could be contacted to be invited to join the study. Evidently, more effective methods are needed for making contact with these women. For example, the SNAP trial [56] proposes asking all pregnant women to complete a form at their ultrasound scan in order to gauge their interest in being recruited for a smoking cessation trial. Many pregnant smokers who report that they have quit smoking are likely to relapse during their pregnancy; therefore recruitment rates might have also been increased by contacting those who said that they had recently quit smoking [57,58]. Additionally, several women were reluctant to join the study as crèche facilities were not available and future studies may need to offer these.

It is concerning that a substantial proportion of women agreed to join the study but failed to attend their first appointment. This may be partly due to the women agreeing to participate over the telephone, having had little time to consider the commitment. Future studies might reduce the number of early drop-outs by offering the women an introduction session prior to joining the study [29]. Our feasibility studies suggest that successful recruitment requires a multi-faceted approach. Telephoning women identified via the hospital database was effective. Advertising through posters and flyers did not appear to boost recruitment rates, nevertheless the latter approaches are easy to implement and forewarn women that they may be telephoned about the study. All the midwives were willing to refer pregnant smokers to the study and several women were recruited in this way, but it is not clear whether these women would have been recruited anyway via telephone contact. Presentations at nuchal scan clinics were relatively labour intensive and did not yield any recruits. This might have been due to the public nature of such presentations, with the smokers being unwilling to identify themselves in such public fora. In summary, our findings suggest that contacting the women by telephone or through their midwife are likely to be the most effective recruitment methods, from which we would expect to recruit around 50 smokers per year from a hospital with 5000 deliveries.

Many women relapse following childbirth [32] and future studies will need to assess whether a physical activity intervention can reduce this postpartum relapse. Studies might also wish to assess levels of smoking reduction, although many confounding variables will apply, and the studies will require careful design. A 50% or more smoking reduction is associated with increased infant birth weight [59] and physical activity may aid smoking reduction [60]. Additionally, it would be interesting to examine the impact of physical activity on peri-natal outcomes such as antenatal complications, duration of labour and birth weight, but the numbers required would be extremely large and such studies may not be feasible.

The women in our studies reported a reduction in the desire to smoke immediately after a bout of supervised exercise, both prior to quitting, and on the quit day following overnight abstinence. This is consistent with findings in non-pregnant smokers [21], and suggests a plausible mechanism through which physical activity might aid smoking cessation. If this can be shown to be a consistent finding, then controlled studies will be needed to assess how long this benefit is sustained after a bout of exercise. The women also reported mood enhancement after a bout of exercise and again this is encouraging, since enjoyment of exercise is likely to increase adherence [61]. This also suggests another avenue of research, since the mood enhancement suggests that the release of neurotransmitters (e.g. endorphins) may be implicated, and biochemical studies could address this issue, and suggest alternative avenues of therapy.

Reports of increases in levels of physical activity tended to be greater in response to the more extensive intervention used in Study 2, than for the Study 1 intervention, and this was especially the case at the final follow-up. Controlled studies with larger samples are required to compare the impact of different intensities of physical activity intervention on activity levels among pregnant smokers. These studies need to include reliable measures for verfying smoking abstinence, such as cotinine. More objective measures of physical activity, such as accelerometers, also need to be employed. Additionally, in order to reduce the possibility of participants providing socially desirable responses future studies should aim to have separate staff deliver the intervention and collect outcomes data. The interview findings suggest that, in general, the women found the physical intervention acceptable and reported many benefits from the intervention. At the outset, the majority of the women reported that they found the idea of regular exercise appealing and that they thought exercising is something that they should do, but only a minority of the women said they saw themselves as the kind of person who exercises regularly. This suggests that the intervention may need to shape the identities of these women towards being someone who is physically active. Future studies may also benefit by tailoring the intervention to accommodate pregnancy related barriers such as fatigue and feeling self-conscious and vulnerable, and these barriers need to be assessed more systematically.

## Conclusion

Our findings suggest that a physical activity intervention is feasible and acceptable as an aid to smoking cessation during pregnancy. A definitive large randomised controlled trial is now required and is under way. If the intervention were shown to be successful for pregnant smokers it would provide a blue print for designing physical activity interventions for other populations of smokers.

## Competing interests

The authors declare that they have no competing interests.

## Authors' contributions

All authors were involved in the various stages of study design. LS liaised with hospital ante-natal services, administered interventions and collected data. MU wrote the paper and all authors commented on drafts and approved the final text.

## Pre-publication history

The pre-publication history for this paper can be accessed here:


